# All on size-coded single bead set: a modular enrich-amplify-amplify strategy for attomolar level multi-immunoassay†

**DOI:** 10.1039/d1sc07048g

**Published:** 2022-02-17

**Authors:** Desheng Chen, Xiaobo Zhang, Liping Zhu, Chenghui Liu, Zhengping Li

**Affiliations:** Key Laboratory of Applied Surface and Colloid Chemistry, Ministry of Education, Key Laboratory of Analytical Chemistry for Life Science of Shaanxi Province, School of Chemistry and Chemical Engineering, Shaanxi Normal University Xi'an 710119 Shaanxi Province P. R. China liuch@snnu.edu.cn; Beijing Key Laboratory for Bioengineering and Sensing Technology, School of Chemistry and Biological Engineering, University of Science and Technology Beijing 30 Xueyuan Road, Haidian District Beijing 100083 P. R. China lzpbd@ustb.edu.cn

## Abstract

Ultrasensitive protein analysis is of great significance for early diagnosis and biological studies. The core challenge is that many critical protein markers at extremely low aM to fM levels are difficult to accurately quantify because the target-induced weak signal may be easily masked by the surrounding background. Hence, we propose herein an ultrasensitive immunoassay based on a modular Single Bead Enrich-Amplify-Amplify (SBEAA) strategy. The highly efficient enrichment of targets on only a single bead (enrich) could confine the target-responsive signal output within a limited tiny space. Furthermore, a cascade tyramide signal amplification design enables remarkable *in situ* signal enhancement just affixed to the target. As a result, the efficient but space-confined fluorescence deposition on a single bead will significantly exceed the background and provide a wide dynamic range. Importantly, the SBEAA system can be modularly combined to meet different levels of clinical need regarding the detection sensitivity from aM to nM. Finally, a size-coded SBEAA set (SC-SBEAA) is also designed that allows ultrasensitive multi-immunoassay for rare samples in a single tube.

## Abstract 2

A modular single bead enrich-amplify-amplify strategy is proposed for simultaneous detection of multiple proteins at the aM level.

## Graphical abstract

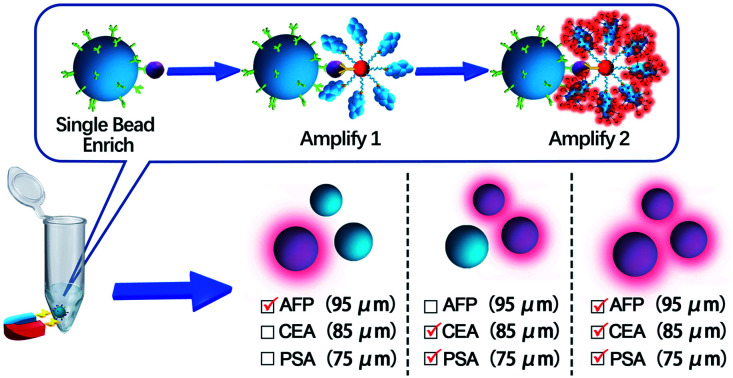

## Introduction

Most diseases originate in minute amounts of tissue or even in a tiny number of cells,^1–3^ and slight fluctuations of protein biomarkers may well reflect the physiological change at the early stages of diseases. Therefore, the sensitive quantification of circulating protein biomarkers is of great significance for early disease diagnosis, postoperative monitoring and precision medicine.^4,5^ Nevertheless, the most challenging issue is that many critical proteins in the blood often range from aM to pM (10^−18^ to 10^−12^ M). Therefore, ultrasensitive protein detection technologies are in high demand.

Traditionally, due to the lack of a self-replication mechanism for proteins, the detection of related substances is mainly based on enzyme-linked immunosorbent assays (ELISA).^6–8^ Despite the convenience that ELISA and its variants have brought to us, clinical applications still urgently require further advances in quantitative protein analysis in sensitivity, dynamic range and multiplex sensing ability with low sample consumption related to limited precious samples.^9–11^ First, in terms of sensitivity, most ELISA-based assays often consume about 100 μL of sample for well-based adsorption and subsequent detection, with the limit of detection (LOD) generally around the pM level.^12,13^ Hence, the concentrations of many clinically important proteins are often near/below the LOD of most ELISA-based assays. More importantly, traditional immunoassays must rely on various signal amplification routes for signal output, always resulting in undesired backgrounds. In other words, if the amplified signal from local targets cannot suppress the cumulative value of the blank background in the whole system, the detection of the associated low concentration samples would be impossible. The dynamic range of protein quantification is another important indicator. Limited by the principle of absorption-based signal output, it is difficult for traditional ELISA-related techniques to achieve rapid analysis with vast concentration spans. Therefore, when performing, for example, allergy analysis and cell cycle-related protein studies that are graded in concentration orders,^14,15^ the available technologies often provide only qualitative or semi-quantitative results.^16,17^ Unfortunately, there has been very little work to overcome the abovementioned problems simultaneously, and most improvements are usually based on trade-offs between the sensitivity and dynamic range, let alone using precious samples for simultaneous testing of multiple components.^18^ As far as we know, elegant digital immunoassays, with Simoa as the most prominent one, provide us with a landmark technique that can efficiently analyze trace proteins across a wide dynamic range.^19–23^ However, the slight drawback is that the digital assay typically requires a specific microfabrication capability and an instrument that is not easily accessible to general laboratories. So, developing a simple technique for precise multi-protein quantification with ultrahigh sensitivity and a wide dynamic range is still urgently expected.

To address the issues mentioned above, herein, we wish to develop a new single bead enrich-amplify-amplify (SBEAA) strategy for ultrasensitive multi-immunoassay. By combining the unique features of highly concentrated target enrichment on a size-coded single bead and highly efficient but space-confined tyramide signal amplification (TSA), the SBEAA strategy has realized simultaneous quantification of multiple antigens at the aM level.

## Results and discussion

**Fig. 1:**
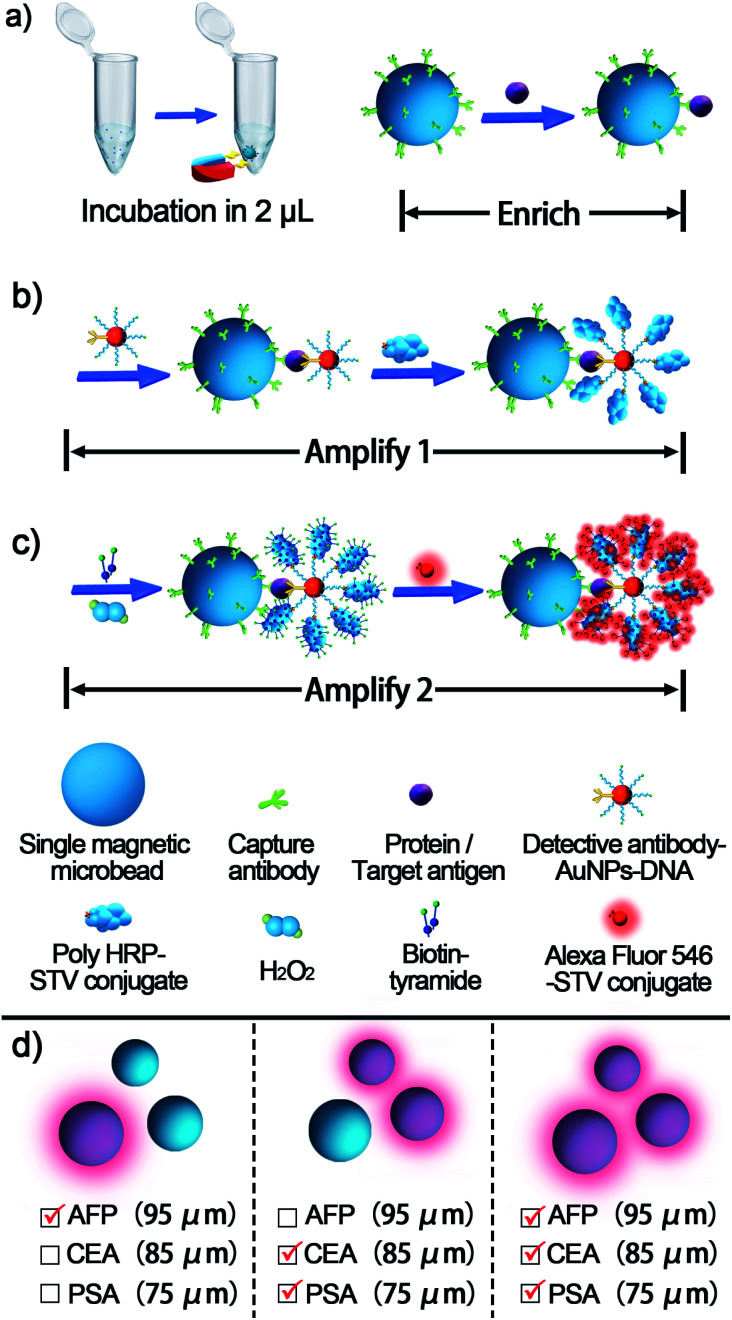
Schematic illustration of the proposed SBEAA and the size-coded single bead set. (a) The mAb1-conjugated single bead can adequately enrich the antigen from as low as 2 μL samples. (b) Illustration of the first level of AuNP-assisted signal amplification (amplify 1), where mAb2 and biotin-DNA co-functionalized AuNPs provide a large amount of terminal biotin for converting a single antigen-binding event into a large amount of biotin-captured STV-poly HRP. (c) The second level of signal amplification (amplify 2). After the loading of STV-poly HRP on the SB, highly efficient TSA can be achieved in the presence of H_2_O_2_ and biotin-tyramide, followed by STV-Alexa Flour 546 staining. (d) Simultaneous detection of PSA, CEA, and AFP antigens using a size-coding mechanism (SC-SBEAA).

The proposed single bead enrich-amplify-amplify (SBEAA) strategy for immunoassay is schematically displayed in Fig. 1 by choosing the clinically significant prostate cancer antigen (PSA) as the model target.^24,25^ As shown in Fig. 1a, the core of the entire strategy is a single NHS-Mag sepharose bead (SB). After conjugating with monoclonal capture antibodies (SB-mAb1) and fine blocking, SB-mAb1 exhibits a low non-specific binding (NSB) effect. Because the diameter of an individual SB is around 80 μm, just a few microliters of the sample (2 μL in this work) are enough for target enrichment and assaying. After sufficient incubation, the antigen is effectively captured (enrich) by SB-mAb1, enabling the concentration of the trace PSA on the SB surface (SB-mAb1-PSA). Assuming that target molecules in a 2 μL reaction system were enriched to a limited space of 2.7 × 10^−4^ μL (around the bead), the strategy ideally achieves a near 7500-fold target concentration. To further expand the detection performance, we then performed a round of gold nanoparticle (AuNP)-amplified tyramide signal amplification (TSA)^26–28^ (amplify 1 and amplify 2) following the single bead enrichment. As illustrated in Fig. 1b, by co-functionalizing AuNPs with the detective antibody (mAb2) and biotinylated DNA, one PSA molecule-mediated immunoreaction will bring an AuNP bearing hundreds of biotin groups onto the SB, which can further capture many STV-poly HRP molecules, the essential initiating enzyme for TSA. Then, in the presence of H_2_O_2_, the poly HRP will catalyze highly efficient TSA, covalently accumulating numerous biotin-tyramide molecules and finally loading a lot of STV-conjugated Alexa-Fluor 546 on the SB (Fig. 1c). As such, each target binding event is successfully converted to significantly amplified fluorescence deposition on the SB *via* the enrich-amplify-amplify strategy (SBEAA). It is worth noting that for TSA, despite its ultrahigh signal amplification efficiency, the enzyme-activated biotin-tyramide deposition can only occur in an *in situ* manner just on protein residues closely surrounding the poly HRP catalysis site, effectively avoiding the undesirable background signals in non-detected areas. Therefore, in the SBEAA, the target PSA as well as the resulting fluorescence signal is highly enriched in the tiny region of the SB surface, and at the same time, the AuNP-assisted TSA with extremely high amplification efficiency and local-confined amplification properties will significantly enlarge the gap between the target-responsive signal and the background signal on the surface of the SB, thereby allowing the detection of extremely low levels of protein. Finally, we employ a size-specific SB for specific antigen detection and refer to these SBs as size-coded single bead sets (SC-SBEAA, Fig. 1d). Eventually, the SC-SBEAA strategy can achieve ultrahigh sensitivity and a wide dynamic range for multiplex immunoassays in a single tube. It is worth noting that in order to guarantee the ultrahigh sensitivity of the SBEAA system, some experimental parameters are carefully controlled to effectively suppress the NSB as well as the background signal, which are described in detail in the ESI.†

**Fig. 2:**
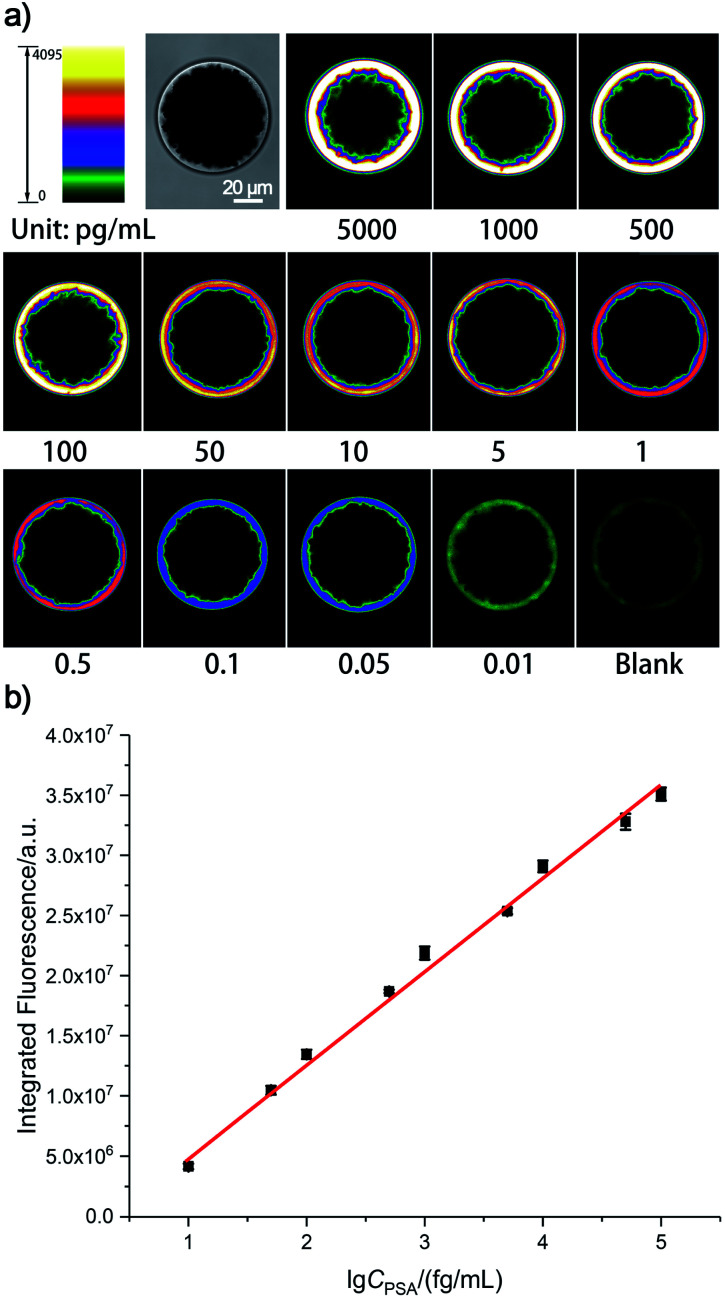
(a) Pseudo-colored fluorescence imaging results of the SBEEA strategy to detect PSA and (b) the relationship between the integrated fluorescence signals of the SB and the PSA concentrations. Error bars represent the standard deviation from three independent measurements.

Ultimately, by using our fluorescence signal acquisition strategy from a single microbead,^29^ the well-designed SBEAA system has achieved reliable detection of PSA down to 10 fg mL^−1^ (Fig. 2a). One can see that the color of the SBs in the pseudo-colored fluorescence images (see the original fluorescence images in Fig. S1†) changes significantly with the change of PSA concentration and can effectively differentiate PSA over more than 5 orders of magnitude from 10 fg mL^−1^ to 5 ng mL^−1^. The results of the integrated fluorescence intensities (FI) of each bead are shown in Fig. 2b, and the fluorescence intensities are linearly proportional to the logarithm of the PSA concentrations in the range from 10 fg mL^−1^ to 100 pg mL^−1^. The correlation equation is FI = 7.24 × 10^6^ lg *C*_PSA_/(fg mL^−1^) −1.57 × 10^6^, with a correlation coefficient (*R*) of 0.9977. After calculation, the LOD of SBEAA can reach an amazing 3.24 fg mL^−1^ (97.2 aM), which is equivalent to 127 PSA protein molecules in a 2 μL system. It is worth noting that the brightness of the SB still increased significantly with the increase of PSA concentration when detecting above 100 pg mL^−1^ PSA. However, with the same data acquisition parameters, due to the strong amplification of SBEAA, the brightness of the SB appeared supersaturated, which exceeded the detection threshold of the microscope. Furthermore, it should be emphasized that although the LOD of the PSA assay in this strategy was very attractive, assaying PSA concentrations lower than 10 fg mL^−1^ showed remarkable signal fluctuations in the results (data not shown). Our interpretation of the phenomenon is that the preparation of diluted specimens far below 10 fg mL^−1^ will no longer follow the statistical law.

**Fig. 3:**
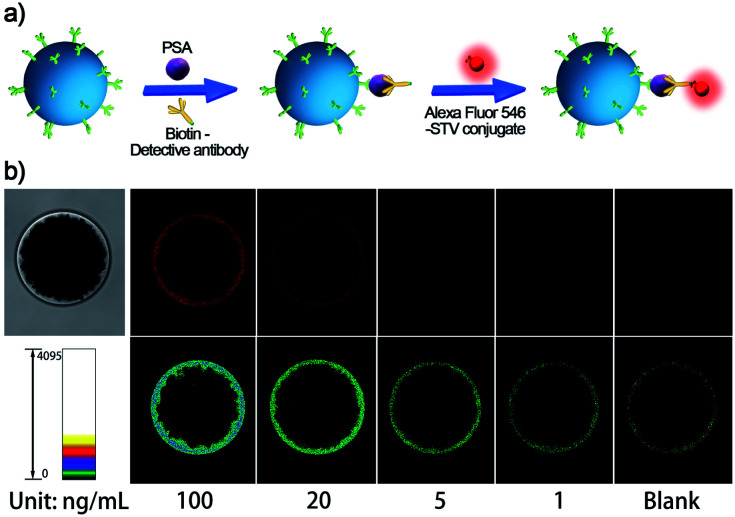
(a) Schematic diagram of the single bead enrichment (SBE) strategy, and (b) its performance in PSA detection. Images in the top panel are fluorescence images of the SB treated with different concentrations of PSA from 0 (blank) to 100 ng mL^−1^. In contrast, the images in the bottom panel illustrate the corresponding SB images by using pseudo-color bars in which different colors indicate different intensities.

As a systemic solution, each of the essential components of the SBEAA strategy is modular and quantifiable. Depending on the sensitivity requirements of clinical applications, different assay solutions can be derived from combining these components (enrich, amplify 1, and amplify 2). The “enrich” operation (SBE) is the first and most central part of SBEAA, and the enrichment effect alone can be illustrated in Fig. 3. After the “enrich” operation, biotin-modified detection antibodies form a classical sandwich structure with SB-mAb1-PSA. With the conversion of PSA information into biotin, the staining using dye-STV conjugate can result in an exportable fluorescent signal (Fig. 3a). Notably, PSA detection down to 1 ng mL^−1^ can be achieved by the SBE without the aid of any signal amplification technique (Fig. 3b, upper panel). We also used pseudo-color to convert the intensity change into color shift (Fig. 3b, down panel), facilitating a more straightforward observation of signal change. The direct sandwich-type testing could detect PSA at the 1 ng mL^−1^ level, close to traditional signal amplification technology-equipped ELISA. Usually, the typical concentration of PSA in human serum is below 4 ng mL^−1^, and when prostate cancer occurs, PSA concentration may become larger than 10 ng mL^−1^. So SBE is clinically sufficient for diagnosing relevant diseases. However, for postoperative low-level PSA monitoring or earlier diagnosis of related diseases (from pg mL^−1^ to ng mL^−1^ range), SBE provides only a basis.

**Fig. 4:**
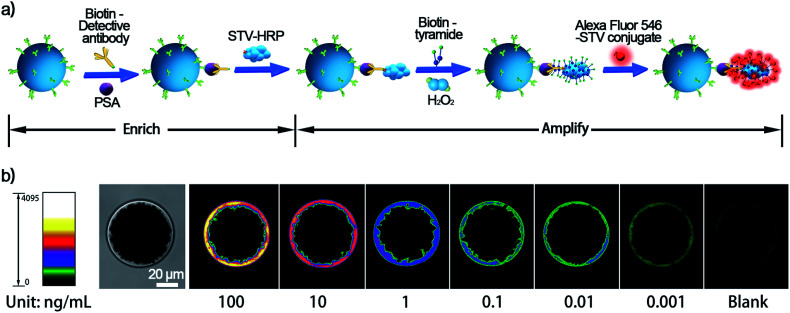
(a) Schematic diagram of the single-bead enrich-amplify (SBEA) strategy by coupling with TSA and (b) its performance in PSA detection ranging from pg mL^−1^ to ng mL^−1^.

Subsequently, an “enrich-amplify” modular design was investigated by using non-AuNP-amplified TSA for protein signal amplification (Fig. 4a). Under optimized TSA parameters (Fig. S2 and S3†), the constructed single-bead enrich-amplify strategy (SBEA) achieved a remarkable improvement in detection sensitivity compared to SBE, which enables PSA detection down to 1 pg mL^−1^ (Fig. 4b). Based on the SBEA imaging data, we integrated the SB fluorescence intensity (FI) at each PSA concentration and performed a linear analysis (Fig. S4†). The correlation equation is FI = 3.80 × 10^6^ lg *C*_PSA_/(ng mL^−1^) + 1.45 × 10^7^, with the LOD at 0.40 pg mL^−1^. Considering that concentrations above 100 ng mL^−1^ are essentially physiologically meaningless, the SBEA system detects up to this and can be considered to possess a linear detection range of nearly 5 orders of magnitude from 1 pg mL^−1^ to 100 ng mL^−1^. Therefore, this level of sensitivity is eligible to meet the task of postoperative PSA monitoring.

According to the literature,^30^ 1 pg mL^−1^ of PSA is equivalent to ∼30 fM, and the number of molecules in a 2 μL system should be at a level of ∼36 000. However, a 1 mm^3^ tumor comprises a million cells, each of which secretes 5000 proteins. When these proteins scatter into 5 L of circulating blood, their concentration translates to about 2 fM.^31^ Moreover, even serum from individuals recently infected with HIV contains 10–3000 virions per mL, resulting in estimated concentrations of the p24 capsid antigen ranging from 50 aM to 15 fM.^32^ Hence, SBEA is fully satisfactory with the most demanding monitoring capabilities. Most importantly, in retrospect, the excellent detection sensitivity of the SBEAA strategy (Fig. 1a–c) could even allow for the single-cell level research.

The specificity of the proposed SBEAA system was investigated by adding different species of proteins, including alpha-fetoprotein (AFP), carcinoembryonic antigen (CEA), human IgG (HIgG), and Goat-anti-human IgG (GaH-IgG), by using the anti-PSA mAb1-conjugated SB and the anti-PSA mAb2. As shown in Fig. S5,† only PSA can induce a significant fluorescence signal on the SB while the responses of other proteins are negligible, clearly suggesting the high specificity of the SBEAA. Moreover, because our method is very sensitive, it allows the use of 10^4^ to 10^5^-fold dilution of the clinical serum sample for PSA detection, which can further efficiently avoid the interference of the complex matrix. Using the SBEAA, the PSA level in the serum sample from a volunteer is determined to be 1.41 ng mL^−1^. Meanwhile, the amount of PSA in the same sample is also tested by the Hospital of Shaanxi Normal University using a commercial ADVIA Centaur® CP PSA Kit, and the determined PSA concentration is 1.62 ng mL^−1^, which agrees well with the detection result from the SBEAA. Such a result indicates that the SBEAA is applicable for PSA analysis in complex samples. To prove the general applicability of the proposed SBEAA, we have further applied it to the detection of CEA and AFP following essentially the same procedures for PSA analysis but using anti-CEA or anti-AFP antibodies. Fluorescence imaging and quantitative analysis results are presented in the ESI.† Their analytical performance is similar to the results of PSA (Fig. S6–S9†) and shows that the SBEEA system has good universality.

**Fig. 5:**
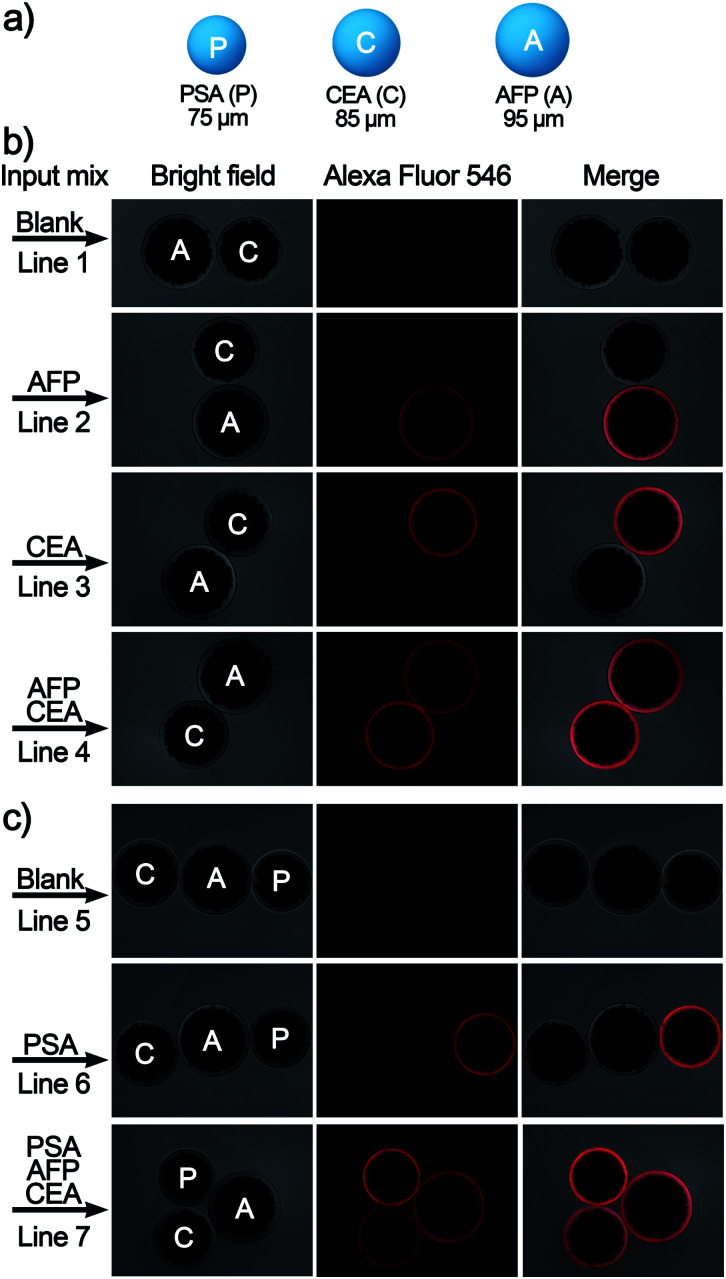
(a) Schematic diagram of the size-coded single bead set for multi-immunoassay of PSA, CEA, and AFP. (b) Imaging results of a two-component assay for AFP/CEA and (c) the corresponding three-component imaging results for detecting AFP/CEA/PSA.

Finally, to meet the potential clinical needs of testing multiple targets in a single sample, we have further developed a new size-encoding mechanism called SC-SBEAA, which can realize the simultaneous detection of multiple antigens in a single reaction. Fig. 1d and 5a illustrate the encoding principle: each SB with a fixed size corresponds to a specific kind of antigen. In this work, the sizes of the AFP-specific SB, CEA-specific SB, and PSA-specific SB were 95 μm, 85 μm, and 75 μm, respectively. In liver cancer and ovarian epithelial tumors, AFP and CEA usually appear simultaneously as cancer markers. Moreover, it is of great significance to improve the accuracy of cancer diagnosis by simultaneous detection. Therefore, we first conducted experiments with simultaneous detection of AFP and CEA, and the experimental results are shown in Fig. 5b. The two SBs had no signal in the absence of any antigen in the input mix (Line 1), and only when one component was present in the input mix, could the corresponding SB be brightened (Lines 2 and 3). Naturally, both SBs were illuminated when both antigens were present (Line 4). Hence, these results demonstrated the feasibility of the encoding strategy for the simultaneous detection of multiple antigens. Furthermore, we extended this strategy to the simultaneous detection of three components, again demonstrating the capability of the SC-SBEAA strategy for multiplex analysis (Lines 5–7).

## Conclusions

In summary, we have proposed a single bead-based “enrich-amplify-amplify” strategy to improve the immunoassay's sensitivity, dynamic range and multiplex assaying capability. In this design, starting from the SBE with only the effect of single bead enrichment, we have built SBEA and SBEAA with different degrees of signal amplification levels. We investigated each system's detection capability and performance, providing a systematic, modular strategy for the relevant antigen/protein detection based on sensitivity requirements. Notably, the SBEAA system achieves the most sensitive detection capability (97.2 aM LOD for PSA) and an ultra-wide dynamic range of up to five orders of magnitude. More fascinatingly, based on a rationally designed size-encoding mechanism (SC-SBEAA), the simultaneous detection of multiple antigens can be accomplished in a single reaction. In regard to real-world clinical applications, we prospect that further integration of SC-SBEAA with microfluidic chips^33^ may provide the most promising and powerful tools for ultrasensitive and multiplexed immunoassay. We envision that if different capture antibody-conjugated single microbeads are packed in a predetermined order/position inside multichannel microfluidic chips, SC-SBEAA can be automatically performed for high-throughput target analysis, which will meet the customized needs of basic research or clinical testing in various scenarios and contribute to human health.

## Data availability

All the relevant data has been provided in the ESI.† We have no additional data to be deposited.

## Author contributions

D. C., C. L. and Z. L. planned and designed the experiments; D. C. completed all experimental operations; X. Z. explored and guided the manipulation of individual microbeads; D. C. and X. Z. analysed the data and wrote the draft; L. Z. has explored the preparation and functionalization of gold nanoparticles; C. L. and Z. L. supervised and coordinated all investigators for this project, provided financial support and revised the manuscript. All authors discussed the results and commented on the manuscript.

## Conflicts of interest

The authors declare no competing financial interests.

## Supplementary Material

SC-013-D1SC07048G-s001
