# Supplementary material for: All on size-coded single bead set: a modular enrich-amplify-amplify strategy for attomolar level multi-immunoassay
Source: Chem Sci. 2022 Feb 17;13(12):3501–6. doi: 10.1039/d1sc07048g (PMC8943839; doi:10.1039/d1sc07048g)
Supplement: SC-013-D1SC07048G-s001 [file SC-013-D1SC07048G-s001.pdf]

Electronic Supplementary Material (ESI) for Chemical Science.
This journal is © The Royal Society of Chemistry 2022

Electronic Supplementary Information

All on Size-Coded Single Bead Set: Modular Enrich-Amplify-Amplify Strategy for Attomolar Level Multi-Immunoassay

Desheng Chen‡,[a,b] Xiaobo Zhang‡,[a] Liping Zhu,[a] Chenghui Liu*[a] and Zhengping Li*[a,b]

[a.] *Key Laboratory of Applied Surface and Colloid Chemistry, Ministry of Education, Key Laboratory of Analytical Chemistry for Life Science of Shaanxi Province, School of Chemistry and Chemical Engineering, Shaanxi Normal University, Xi'an 710119, Shaanxi Province, P. R. China. E-mail: liuch@snnu.edu.cn (C. Liu)*

[b.] *Beijing Key Laboratory for Bioengineering and Sensing Technology, School of Chemistry and Biological Engineering, University of Science and Technology Beijing, 30 Xueyuan Road, Haidian District, Beijing 100083 P. R. China. E-mail: lzpbd@ustb.edu.cn (Z. Li)*

Table of Contents

1. Reagents and Materials

2. Preparation of mAb2-AuNPs-biotin DNA

3. Preparation of capture antibody-conjugated SB

4. Standard Procedures for the SBE strategy

5. Standard Procedures for the SBEA strategy and SBEAA strategy

6. The original fluorescence imaging results of the SBEAA strategy for PSA detection

7. Optimization of the concentration of TSA reagent for SBEA

8. Optimization of the TSA reaction time for SBEA

9. The linear relationship between integrated fluorescence intensities (FI) of the beads and the logarithm of the PSA concentrations by using SBEA

10. Procedures of the single bead-based size encoding strategy for the simultaneous detection of PSA/AFP/CEA

11. Evaluation of the specificity of the SBEAA

12. Evaluation of the generality of the SBEAA for the detection of CEA and AFP

1. Reagents and Materials

PSA, anti-PSA monoclonal capture antibody (mAb1), anti-PSA monoclonal detective antibody (mAb2), biotinylated anti-PSA monoclonal detective antibody, human alpha-fetoprotein antigen (AFP), anti-AFP monoclonal capture/detective antibodies, Human carcinoembryonic antigen (CEA), monoclonal capture/detective anti-CEA antibodies were purchased from Linc-Bio Science (Shanghai, China). In addition, human IgG (HIgG) and Goat-anti-human IgG (GaH IgG) were obtained from Sanchen Biotechnology (Nanjing, China).

The Mag Sepharose microbeads, composed of iron oxide cores embedded in agarose matrices and are surface-functionalized with N-hydroxysuccinimide (NHS), were purchased from GE Healthcare. Thermo Fisher Scientific supplied the Alexa Fluor 546-STV (AF546-STV) conjugates, and Streptavidin conjugated Poly HRP (STV-Poly HRP). Tween-20 and bovine serum albumin (BSA) were acquired from Sangon Biotech (Shanghai, China). The alkylthiol-capped 5'-Biotin oligonucleotides probe (5'-Biotin-TTTTTTTTTTTTTTTTTTTTTTTTT-SH-3') was synthesized and purified by Sangon Biotech (Shanghai, China). Gold (III) chloride trihydrate was acquired from Sigma-Aldrich. TSA Biotin Reagent Pack (containing biotin-tyramide and 1× TSA buffer) was purchased from Beijing Biodragon Immunotechnologies Co., Ltd. All of the other chemical reagents were of analytical grade and used as received without further purification.

The detailed components of the used buffers in this study are listed below:

1× PBS (10 mM, pH 7.4, containing 137 mM NaCl and 2.7 mM KCl);

PBS-BSA (1× PBS with 1% BSA and 0.1% tween-20);

PBST (1× PBS with 0.05% tween-20);

Blocking buffer A (0.5 M ethanolamine, 0.5 M NaCl, pH 8.3);

Blocking buffer B (0.1 M Na-acetate, 0.5 M NaCl, pH 4.0);

1× TSA buffer (0.01 M PB，0.15 M NaCl，0.01% H_2O_2，pH 7.4).

2. Preparation of mAb2-AuNPs-biotin DNA

The 16 nm colloidal AuNPs were prepared following the well-established citrate-reduction method. The mAb2-AuNPs-biotin DNA nanocomplexes were prepared according to a modified literature protocol.[S1] Typically, 1 mL of 16 nm colloidal AuNPs were adjusted to pH 9.2 - 9.5 using a 0.1 M Na_2CO_3 solution. Then, 10 μL anti-PSA mAb2 (1.0 mg/mL) was introduced, and the mixture was incubated at room temperature for 20 min with mild rolling. Afterward, the alkylthiol-capped 5'-Biotin oligonucleotides (1 nmol) were pipetted into the mixture. After gently shaking for 5 min, the mixture was further incubated at 4 °C overnight. Following that, the mixture was first buffered to 10 mM PB (pH 7.2), and then the NaCl concentration was tuned to 0.15 M under slow stirring. The mAb2-AuNPs-biotin DNA was purified by multiple rounds of centrifugation. Finally, the obtained mAb2-AuNPs-biotin DNA was dispersed in 1 mL of PBS-BSA and stored at 4 °C. The pre-treatment of the mAb2-AuNPs-biotin DNA with BSA as well as tween-20 can efficiently suppress their non-specific binding effect.

3. Preparation of capture antibody-conjugated SB

Typically, hundreds of SBs contained in 80 μL PBST were deposited onto a

transparent 96-well plate cover hole to assist the capture of individual SBs. A micromanipulator system (Narishige) equipped on an Olympus IX53 inverted microscope with a monitor is used to manipulate a SB. With the help of the camera and monitor, we can search the SBs with suitable sizes on the screen with naked eyes. A homemade ruler is used to help us select the beads with the desired narrow size (80 ± 5 μm). Once a desirable SB is selected, we only need a pipette to catch and transfer it into a tube. More detailed manipulations of pipetting microbeads have been described in our group's previous work.[S2,S3] We first select 20 uniform microbeads together in a tube, and add 100 μL ice-cold 1 mM HCl to activate their NHS surfaces. Next, after isolation, add the mAb1 (0.1 μg) solution into the processed beads and mix well, and let the medium incubate with mild shaking for 1 h. Then the active site on the surface of the microbeads are effectively blocked by using Blocking buffer A and B in turn for three times according to the product manual. Then, we added 200 μL PBST to wash the microbeads (pipette and drain out the washing buffer PBST for 3 times and then the PBST is removed by magnetic separation), finally the mAb1-conjugated microbeads are stored in PBS-BSA. Immediately before the immunoassay, the uniformly modified SBs are transfered into each reaction tube one by one.

It should be noted that since both the surfaces of mAb1-conjugated SBs and the mAb2/biotin-DNA-functionalized AuNPs are passivated with BSA, and BSA/Tween-20 are also added in the immunoreaction system, the nonspecific interaction between the SB and the AuNPs can be efficiently suppressed.

4. Standard procedures for the SBE strategy

The SB-mAb1 was incubated with 1 μL of PBS-BSA containing series dilutions of target antigen for 1 h. Moreover, 2 μL of biotinylated anti-PSA detective antibody (0.1mg/mL) was added to perform the noncompetitive sandwich immunoreaction at room temperature for another 1 h under mild shaking. After washing the superfluous detective antibody, 5 μL of excess AF546-STV (0.1 μg) in PBS was added for fluorescence staining. After 1h, the SB was washed and immediately subjected to fluorescence imaging.

5. Standard procedures for the SBEA strategy and SBEAA strategy.

The immunoreaction part of the SBEA strategy was the same as the SBE Strategy. After washing the excess detective antibody, excess streptavidin Poly HRP (0.1 μg) was added into the bead. After 1 h incubation, wash the bead and prepare for TSA reaction.

Mix 50 μL 1× TSA buffer (containing 0.01% H_2O_2) and 1 μg biotin-tyramide (1 μL from 1 mg/mL stocking solution) together, then dilute the mixture 10 times by PBS. The 10 μL TSA reaction solution was added to each SB and mild shake for 30 min. After washing, adding 5 μL of excess AF546-STV (0.1 μg) for fluorescence staining. After 1h, the SB was washed and immediately subjected to fluorescence imaging.

The only difference before TSA reaction between SBEAA strategy and SBEA strategy is replacing the biotinylated anti-PSA detective antibody with 2 μL mAb2-AuNPs-biotin DNA in the immunoreaction part. Since the sensitivity of SBEAA was further remarkably improved, we also optimized the reaction conditions for TSA reaction part of SBEAA. Finally for TSA amplification in SBEAA, mix 50 μL 1×

TSA buffer and 2 μg biotin-tyramide together, then dilute the mixture 50 times by PBS, add 10 μL TSA reaction solution to each SB and mild shake for 30 min. The next steps are also the same as SBEA.

All fluorescence images were taken on an Olympus FV-1200 laser scanning confocal microscope following modified protocols in our previous report.[S2,S3] Briefly, the SB was spread on a coverslip, and its fluorescence image was obtained. By collecting the fluorescence at test parameters for AF546 of the instrument. The integrated fluorescence intensity of each SB was acquired for the quantitative analysis of the target antigen. It should be noted that the maximum fluorescence value of a bright spot that the fluorescence microscope can quantitatively acquire is 4096. Therefore, to acquire a brighter MB image but not exceed this maximum value, the PMT voltage of the fluorescence microscope may be reasonably tuned for the imaging of MBs at different batches for different experimental conditions.

6. The original fluorescence imaging results of the SBEAA strategy for PSA detection

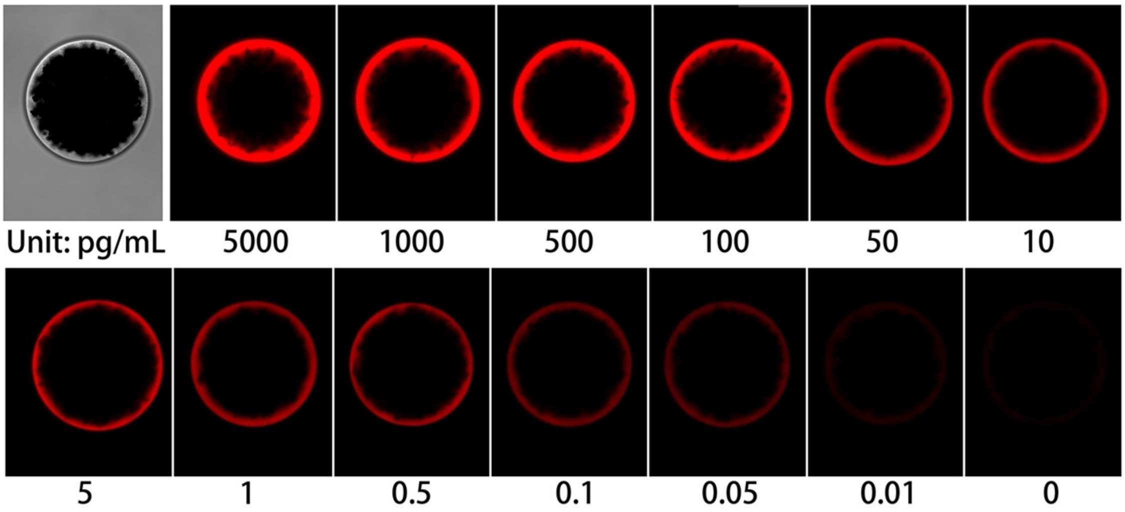

Fig. S1 The original fluorescence imaging results of the SBEAA strategy for PSA

detection. In order to display the results more intuitively, the corresponding pseudo-color results (displayed in different colors for different intensities) is showed in Fig. 2 in the main text.

7. Optimization of the concentration of TSA reagent for SBEA

According to the instruction of the commercial TSA Biotin Reagent Pack, the recommended TSA reagent for traditional biological staining is mixing 50 μL 1× TSA buffer (containing 0.01% H_2O_2) and 1 μg biotin-tyramide together. Herein, to reduce the nonspecific background signal, so as to achieve the best signal-to-noise ratio in the SBEA, we further optimized the concentration of the reaction mixture on PSA detection. The reaction mixture was undiluted or was diluted 10 times, 50 times, 100 times, or 200 times with PBS buffer to detect 50 pg/mL PSA.

The concentration of TSA reagent has a significant impact on the single bead-based assay. The PSA-induced signal is strong under the high concentration of the reaction solution, while the corresponding blank value is also very high. Conversely, the sample signal is weak under the low concentration of the reaction solution, and the relative blank value is also low. However, due to the limitation of instrument range, it is impossible to accurately measure the fluorescence intensity of each group of experiments under the same test conditions. Therefore, we adjusted the PMT test voltage of the microscope to ensure that the integrated fluorescence intensities of each group of blank controls were almost the same. So that all test results were guaranteed within the detectable range. The difference between the signal intensity and the blank of the sample is then compared to evaluate the experimental conditions. The test

voltage for undiluted, diluted 10 times, 50 times, 100 times, 200 times samples used in order: 350V, 380V, 410V, 460V, 480V. The experimental results are shown in Fig. S2. It was found that when the TSA reaction mixture was diluted 10 times by the PBS buffer, the difference between the integrated fluorescence intensity of the sample signal and the blank signal was the maximum. Therefore, we finally determined that the TSA reaction mixture should be diluted 10 times with PBS buffer. So in the final reaction condition, tyramide concentration is 2 μg/mL with 0.001% H_2O_2.

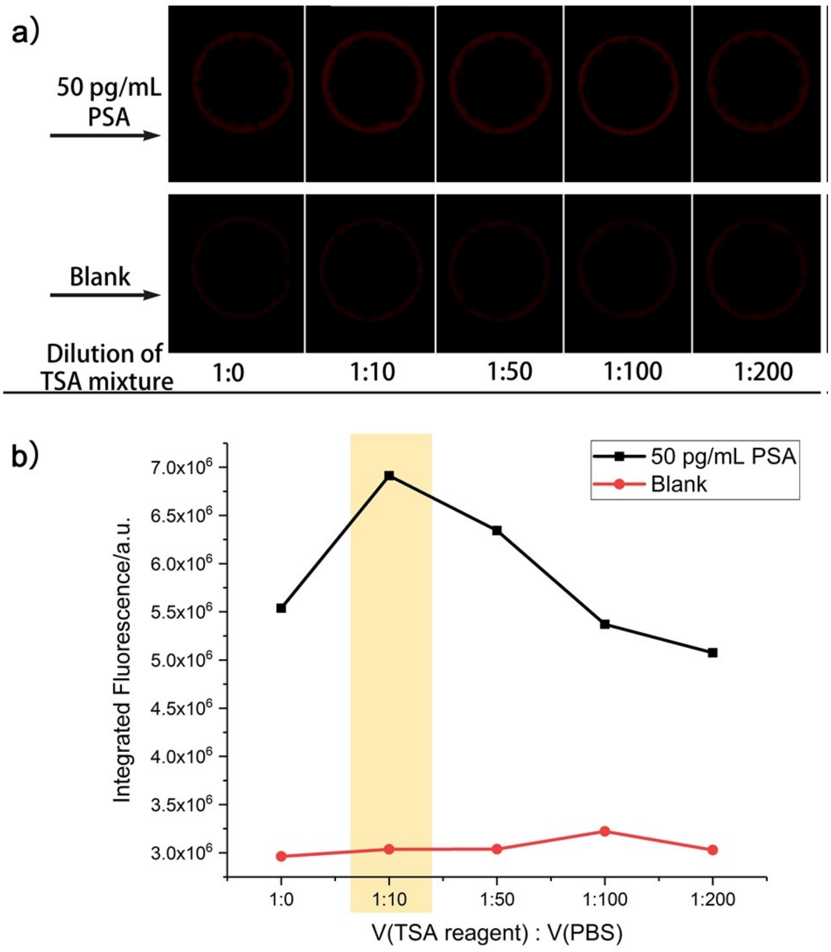

Fig. S2 Optimization of the concentration of TSA reagent for the SBEA.

8. Optimization of the TSA reaction time for SBEA

We also optimized the TSA reaction time. The experimental results at 10 min, 20 min, 30 min, 40 min, and 50 min of TSA reaction were tested. Likewise, we adjusted the test voltage so that the integrated fluorescence intensities of each group of blank controls was almost the same. The difference between the signal intensity and the blank of the sample is then compared to evaluate the experimental conditions. The test results are shown in Fig. S3. It can be seen that the difference between the experimental signal and the blank signal reaches the maximum at 30 min, so we chose the TSA reaction time of 30 min as the optimal condition for subsequent experiments.

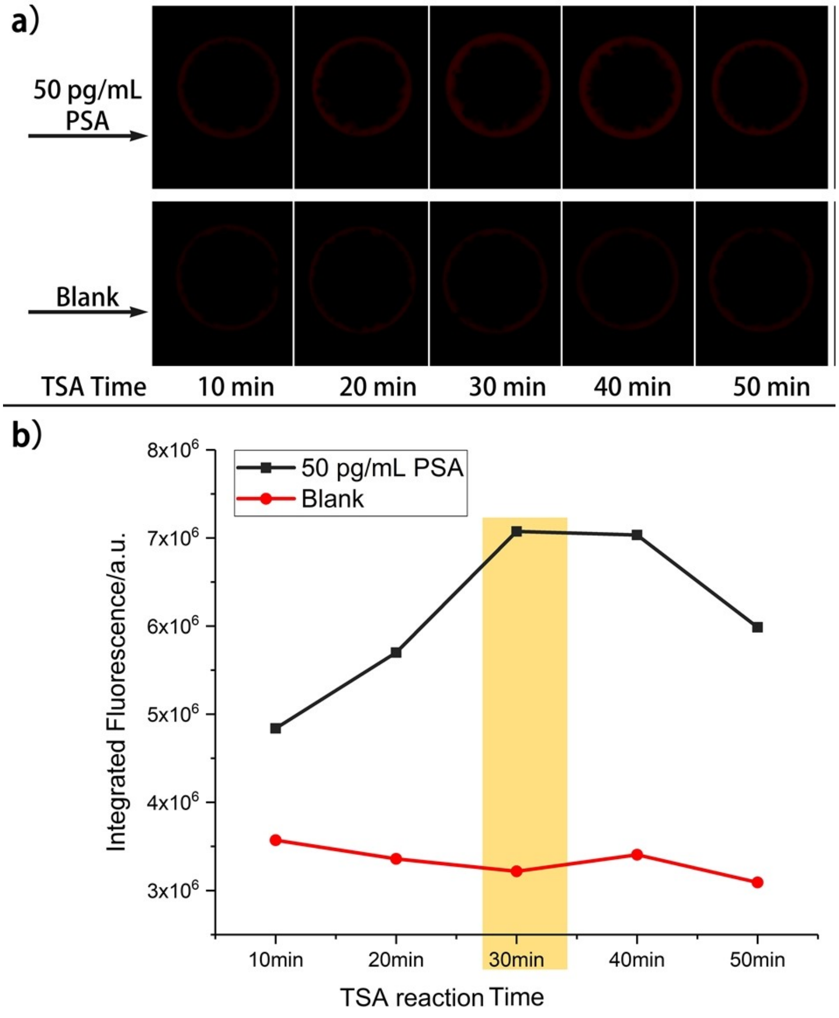

Fig. S3 The optimization of TSA reaction time for SBEA.

9. The linear relationship between integrated fluorescence intensities (FI) of the beads and the logarithm of the PSA concentrations by using SBEA

We Integrate the fluorescence signals of each bead image for the quantitative analysis of the target PSA in the SBEA. As shown in Fig. S4, the beads' integrated fluorescence intensities (FI) are linearly proportional to the logarithm of the PSA concentrations in the range from 1 pg/mL to 100 ng/mL. The correlation equation is $FI = 1.45 \times 10^6 + 3.80 \times 10^6\ lgC_{PSA}/(ng/mL)$, with a correlation coefficient (R) of 0.9973.

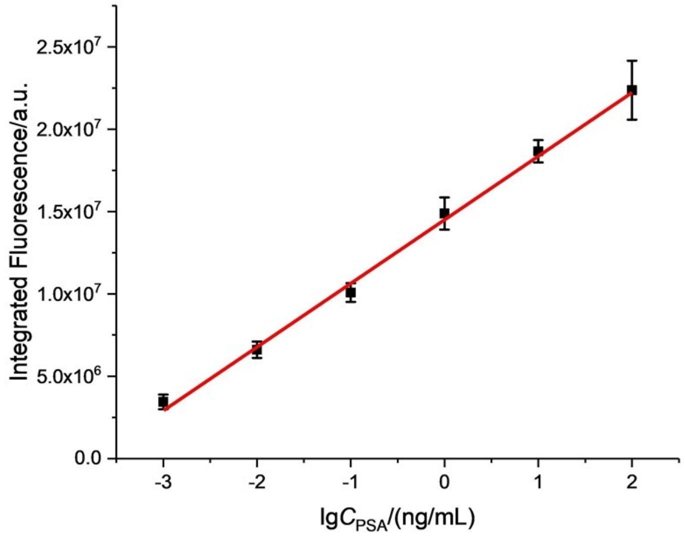

Fig. S4 The relationship between the integrated fluorescence signals of the SBs and the PSA concentrations. Error bars represent the standard deviation from three independent measurements.

10. Procedures of the single bead-based size encoding strategy for the simultaneous detection of PSA/AFP/CEA

Three kinds of single microbead of different sizes were selected respectively. Conjugating AFP antibodies on the 95 μm microbeads, 85 μm microbeads for CEA,

and 75 μm microbeads for PSA, respectively. After such microbeads have been modified, blockeded, and washed respectively, one of each of the three types of microbeads is put into a single reaction tube together. After introduction of the samples containing different combinations of antigens, the three types of SBs can only enrich a correspondingly specific antigen after 1 h immunoreaction. Then, after further incubation with a cocktail solution containing three types of mAb2-AuNPs-biotin DNA for anoher 1 h, the three target-encoded SBs are subjected to TSA amplification and fluorescence imaging simultaneously. The following experimental procedures was the same as that for a single microbead-based SBEAA assay.

11. Evaluation of the specificity of the SBEAA

The specificity of the proposed SBEAA is interrogated by challenging the SBEAA system with different protein species, including AFP, CEA, human IgG (HIgG), and Goat-anti-human IgG (GaH-IgG), by using the anti-PSA capture antibody-conjugated SB and the anti-PSA detective antibody-AuNPs-biotin DNA. As shown in Fig. S5, only PSA can arouse a significant fluorescence signal on the SB while the responses of other proteins are negligible, clearly suggesting the high specificity of the SBEAA.

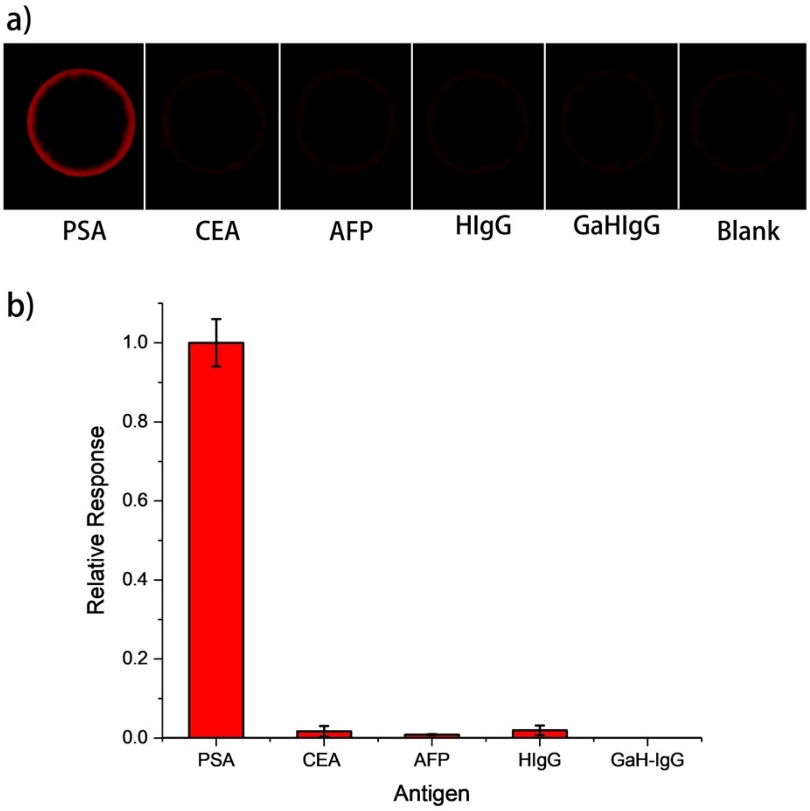

Fig. S5 Specificity evaluation of the SBEAA. Fluorescence images of the SBEAA system were acquired in the presence of different antigens by using anti-PSA antibodies. The concentrations of all of these antigens are 10 pg/mL.

12. Evaluation of the generality of the SBEAA for the detection of CEA and AFP

We changed the types of antibodies conjugated with SB and AuNPs and examined the universality of SBEAA using CEA and AFP detection as an example. The results of CEA testing are shown in Fig. S6 and S7. The integrated fluorescence of SBs are proportional to the CEA concentrations in the range from 50 fg/mL to 50 pg/mL with the correlation equation FI = $-1.69 \times 10^6 + 6.10 \times 10^6$ $lgC_{CEA}/(fg/mL)$ and a correlation coefficient (R) of 0.9966.

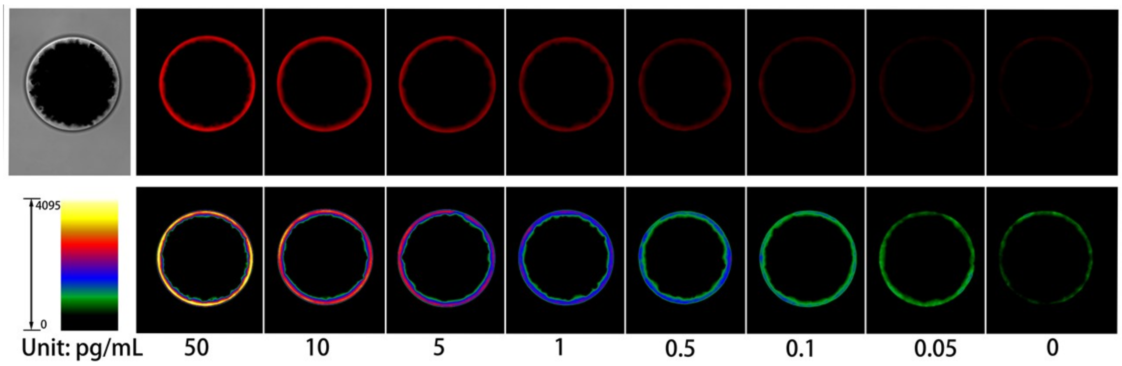

Fig. S6 The fluorescence images of the SBs for different concentrations of CEA from 0 (blank) to 50 pg/mL. It can be seen from the experimental results in the image that the signal of 50 fg/mL CEA can be distinguished from the blank signal. The images in the top panel are raw fluorescence imaging. The panel's bottom illustrates the corresponding SB images using pseudocolor bars in different colors to indicate different intensitieslors.

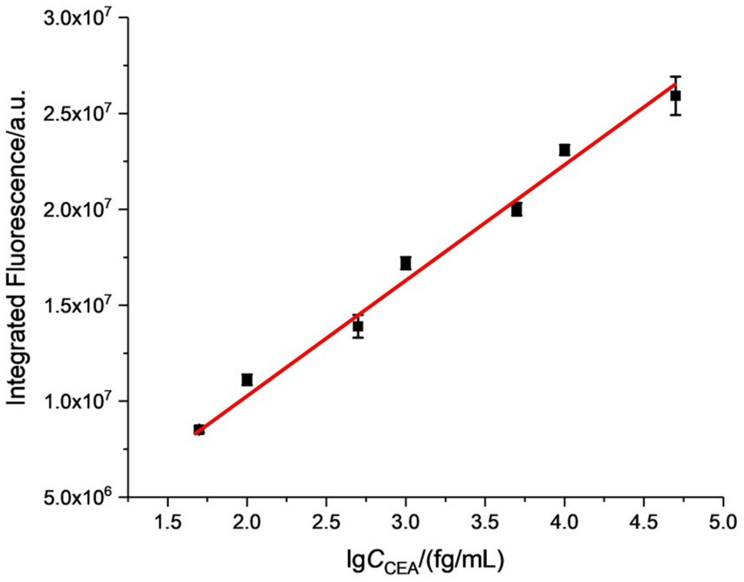

Fig. S7 The relationship between the integrated fluorescence signals of the SB and the CEA concentrations of SBEAA from 0 (blank) to 50 pg/mL. Error bars represent the standard deviation from three independent measurements.

Meanwhile, as shown in Fig. S8 and S9, AFP also showed similar test results. There is a good linear relationship between the integrated fluorescence of SBs and logarithm of AFP concentrations from 10 fg/mL to 50 pg/mL with the correlation equation FI = $5.00 \times 10^5 + 7.21 \times 10^6$ lgC_{AFP}/(fg/mL) and a correlation coefficient (R) of 0.9900. These results demonstrate that by employing the corresponding target-

specific antibodies, the proposed SBEAA can be readily extended as a general strategy for detecting various antigens.

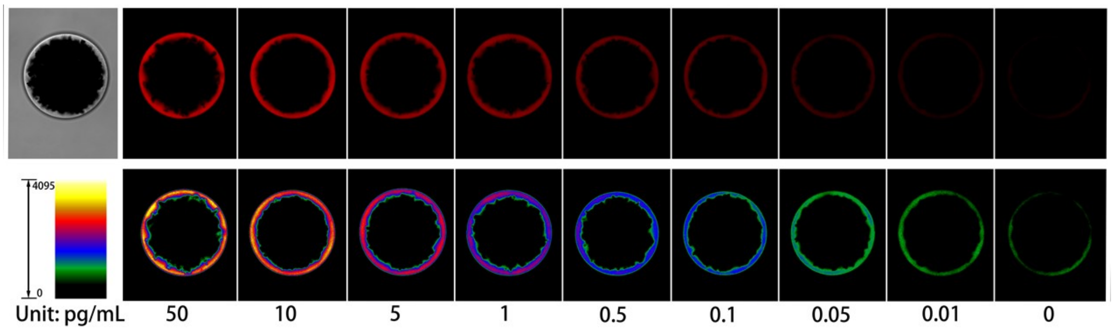

Fig. S8 The fluorescence images of the SBs for different concentrations of AFP from 0 (blank) to 50 pg/mL. It can be seen from the experimental results in the image that the signal of 10 fg/mL AFP can be distinguished from the blank signal. The images in the top panel are raw fluorescence imaging, in the bottom panel are illustrations of the corresponding SB images by using pseudocolor bars in which different colors indicate different intensities.

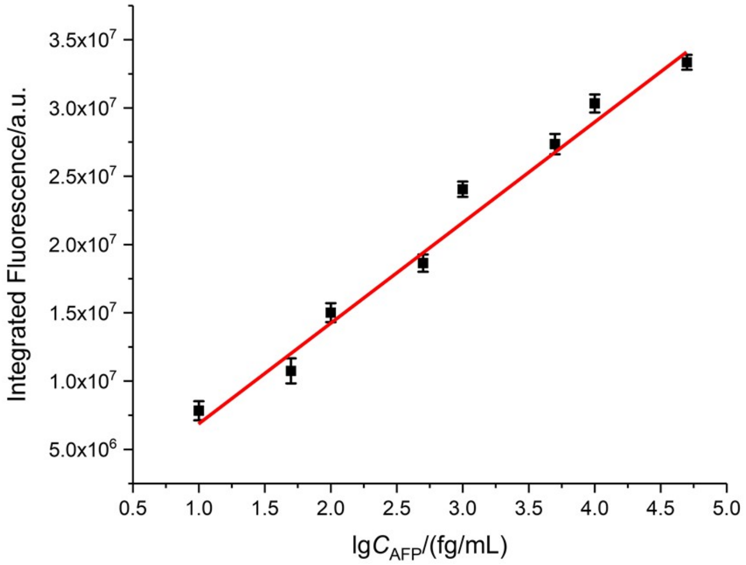

Fig. S9 The relationship between the integrated fluorescence signals of the SB and the AFP concentrations of SBEAA from 0 (blank) to 50 pg/mL. Error bars represent the standard deviation from three independent measurements.

References

[S1] S. I. Stoeva, J. S. Lee, J. E. Smith, S. T. Rosen, C. A. Mirkin, *J. Am. Chem. Soc.*,

2006, **128**, 8378-8379.
[S2] X. Zhang, C. Liu, H. Wang, H. Wang, Z. Li, *Angew. Chem. Int. Ed.*, 2015, **54**, 15186-15190.
[S3] X. Zhang, C. Liu, L. Sun, X. Duan, Z. Li, *Chem. Sci.*, 2015, **6**, 6213-6218.
